# Recent advances in cardiorespiratory monitoring in acute respiratory distress syndrome patients

**DOI:** 10.1186/s40560-024-00727-1

**Published:** 2024-05-05

**Authors:** Davide Chiumello, Antonio Fioccola

**Affiliations:** 1 Department of Health Sciences, University of Milan, Milan, Italy; 2 Department of Anesthesia and Intensive Care, ASST Santi Paolo e Carlo, San Paolo University Hospital Milan, Via Di Rudinì 9, Milan, Italy; 3 Coordinated Research Center on Respiratory Failure, University of Milan, Milan, Italy; 4 Department of Health Sciences, University of Florence, Florence, Italy

**Keywords:** Acute respiratory distress syndrome, Ventilator-induced lung injury, Oxygen delivery, Fluid therapy

## Abstract

**Background:**

Recent advances on cardiorespiratory monitoring applied in ARDS patients undergoing invasive mechanical ventilation and noninvasive ventilatory support are available in the literature and may have potential prognostic implication in ARDS treatment.

**Main body:**

The measurement of oxygen saturation by pulse oximetry is a valid, low-cost, noninvasive alternative for assessing arterial oxygenation. Caution must be taken in patients with darker skin pigmentation, who may experience a greater incidence of occult hypoxemia. Dead space surrogates, which are easy to calculate, have important prognostic implications. The mechanical power, which can be automatically computed by intensive care ventilators, is an important parameter correlated with ventilator-induced lung injury and outcome. In patients undergoing noninvasive ventilatory support, the use of esophageal pressure can measure inspiratory effort, avoiding possible delays in endotracheal intubation. Fluid responsiveness can also be evaluated using dynamic indices in patients ventilated at low tidal volumes (< 8 mL/kg). In patients ventilated at high levels of positive end expiratory pressure (PEEP), the PEEP test represents a valid alternative to passive leg raising. There is growing evidence on alternative parameters for evaluating fluid responsiveness, such as central venous oxygen saturation variations, inferior vena cava diameter variations and capillary refill time.

**Conclusion:**

Careful cardiorespiratory monitoring in patients affected by ARDS is crucial to improve prognosis and to tailor treatment via mechanical ventilatory support.

## Keypoints

1. The SpO_2_/FiO_2_ ratio, corrected minute ventilation and ventilatory ratio are valid surrogates for estimating gas exchange in ARDS patients, and caution should be taken in patients with darker skin pigmentation and moderate–severe ARDS.
2. Changes in esophageal pressure during noninvasive respiratory support and mechanical power must be carefully monitored to estimate PSILI and VILI in ARDS patients undergoing mechanical ventilation.
3. The use of dynamic indexes of fluid responsiveness should be encouraged in ARDS patients. Pulse pressure variation and stroke volume variation have also been validated in mechanically ventilated patients with low tidal volume.
4. A possible alternative to passive leg raising in a mechanically ventilated patient is the PEEP test. Two minimally invasive alternatives to predict fluid responsiveness are changes in central venous oxygen saturation and capillary refill time after a passive leg raising or a fluid challenge.

## Introduction

Patients with acute respiratory distress syndrome (ARDS) exhibit inflammatory pulmonary edema resulting from changes in endothelial and epithelial permeability, leading to organ damage. The severity of ARDS determines the application of different types of mechanical support. Superimposed hemodynamic impairment may complicate patient management, worsening outcomes. Therefore, a comprehensive evaluation of ARDS patients involves careful respiratory and hemodynamic monitoring, encompassing both invasive and noninvasive technologies, along with clinical and laboratory data. This approach is crucial for tailoring therapeutic strategies to individual patients and minimizing lung injury.

**Fig. 1:**
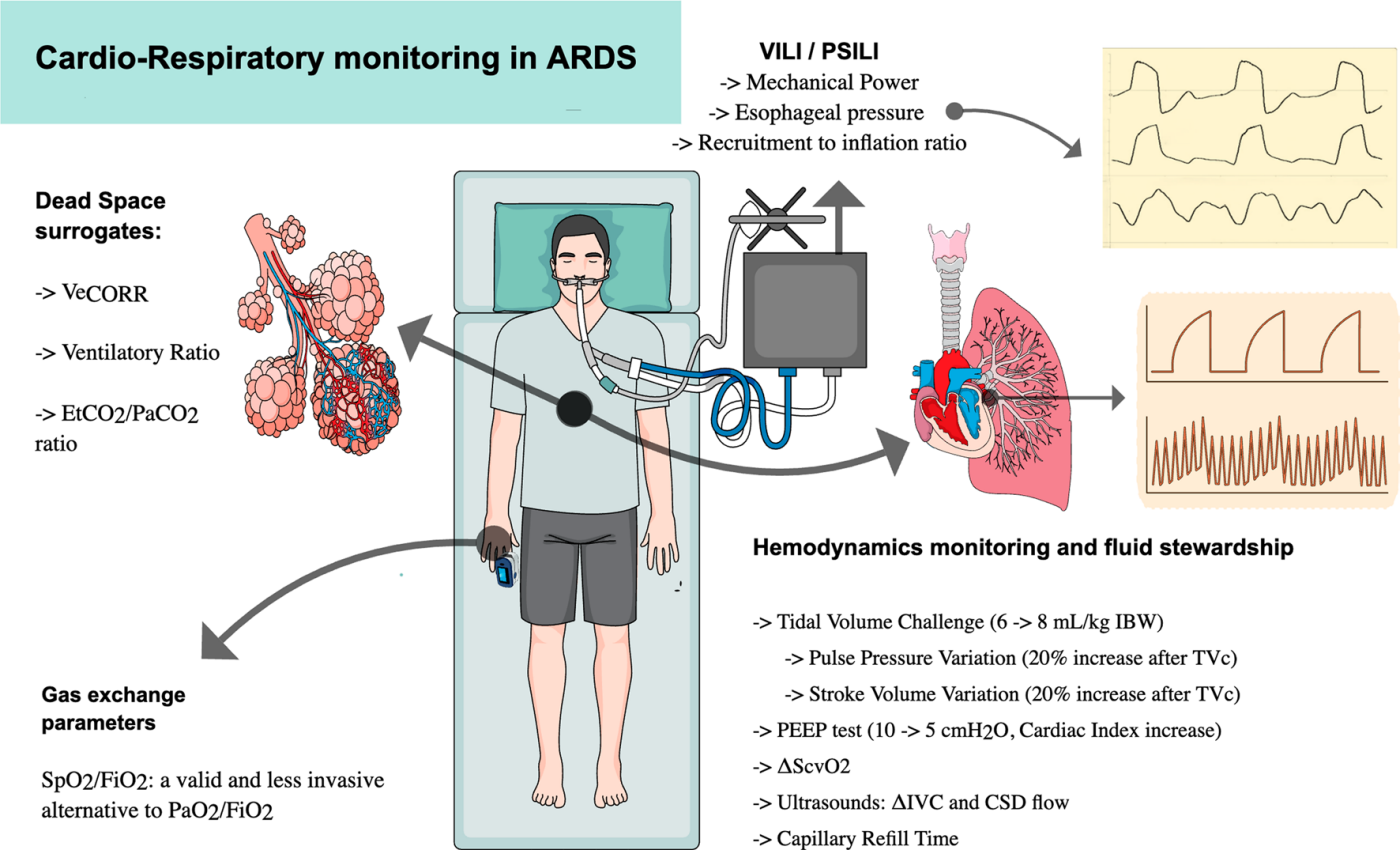
Respiratory and hemodynamic monitoring in patients affected by ARDS. Recent evidence about respiratory and hemodynamic monitoring in mechanically ventilated patients is available. *Ve*_*CORR*_ corrected minute ventilation, *EtCO*_*2*_ end-tidal CO_2_, *SpO*_*2*_ peripheral oxygen saturation, *FiO*_*2*_ fraction of inspired oxygen, *PaO*_*2*_ arterial oxygen partial pressure, *mL* milliliters, *kg* kilograms, *IBW* ideal body weight, *TVc* tidal volume challenge, *PEEP* positive end expiratory pressure, *ΔScvO*_*2*_ central venous oxygen saturation increase

This manuscript reviews strategies for respiratory and hemodynamic monitoring in ARDS patients, highlighting the most recent data and clinical utility in daily management, as synthesized in Fig. 1.

### Respiratory monitoring

Careful respiratory monitoring is essential in patients affected by ARDS. This approach allows the application of an adequate intensity of treatment and reduces injuries caused by mechanical ventilation (MV).

### Gas exchange efficiency

Gas exchange is directly affected by pulmonary alterations induced by ARDS. In this section, we review the renewed role of pulse oximetry and useful surrogate indices of dead space.

#### Pulse oximetry

Pulse-oximetry exploits the principle of spectrophotometry to quantify the amount of oxygenated hemoglobin in blood, allowing continuous noninvasive monitoring of arterial saturation [1]. The difference between arterial oxygen saturation (SaO_2_) measured via blood gas analysis and oxygen saturation measured via pulse-oximetry (SpO_2_) is normally less than 3% [2]. However, the accuracy of SpO_2_ may be lower among patients with darker skin pigmentation, thus overestimating arterial oxygen saturation. This phenomenon, as recently demonstrated by Henry et al*.*, possibly increases the incidence of occult hypoxemia, i.e., patients in which SaO_2_ is lower than 88% with an SpO_2_ higher than 92% [3]. The clinical consequences of occult hypoxemia have also been investigated during the recent pandemic. In COVID-19 patients, occult hypoxemia is more frequent in Asian, Black and non-Black Hispanic patients than in White patients, with lower treatment eligibility for these three ethnicities [4].

The ratio of pulse-oximetric oxygen saturation to the fraction of inspired oxygen (SpO_2_/FiO_2_) is an acceptable surrogate of the ratio of the partial pressure of arterial oxygen to FiO_2_ (PaO_2_/FiO_2_). Its use has been described both in invasively and noninvasively ventilated patients [5–9]. The SpO_2_/FiO_2_ ratio is a good outcome predictor both in patients with coronavirus disease (COVID-19) and non-COVID-19 ARDS patients [10, 11]. In patients with COVID-19-associated pneumonia requiring oxygen therapy, the SpO_2_/FiO_2_ ratio at admission showed an area under the curve (AUC) of 85% for the prediction of ARDS occurrence [12]. Kim et al*.* showed that the SpO_2_/FiO_2_ ratio can predict high-flow nasal cannula (HFNC) failure [13]. Moreover, SpO_2_/FiO_2_ shows a good correlation with PaO_2_/FiO_2_ in invasively ventilated COVID-19 ARDS patients, and when computed on day 2 and day 3, it is associated with outcome [11]. These data confirm the reliability of pulse oximetry for evaluating gas exchange in ARDS patients and for following this trend, as pulse oximetry is continuously measurable. It is easy to measure and is thus especially valid in contexts in which a blood gas analyzer is not promptly available.

The optimal SpO_2_ concentration for ARDS treatment is still a matter of debate, ranging from 88% to 96–100% to balance the risk of hyperoxia and hypoxia. In a recent large randomized controlled trial (RCT), Semler et al*.* showed that, in mechanically ventilated patients, the use of a lower (90%, range from 88 to 92%), intermediate (94%, 92–96%) or higher (98%, 96–100%) SpO_2_ target does not affect either ventilator-free days or hospital outcomes [14].

#### Dead space

Physiological dead space is the inspired volume of air that does not participate in gas exchange. It includes anatomic and alveolar dead space [15]. In mechanically ventilated patients, the anatomic dead space remains relatively constant, while the alveolar dead space can significantly increase according to alterations in the ventilation/perfusion (*V*/*Q*) ratio [16, 17].

In a seminal study, Nuckton et al. demonstrated that physiological dead space is significantly higher in non-ARDS survivors than in survivors [18]. Like in ARDS patients, COVID-19 pneumonia is characterized by an increase in minute ventilation and an increase in the dead space fraction [19, 20]. Additionally, in COVID-19 ARDS patients, there is a significant association between the amount of dead space computed in the first 7 days and mortality [21]. According to a secondary analysis of the PRoVENT COVID-19 study, the dead space fraction is significantly greater in nonsurvivors and increases more during the first four days than in survivors, suggesting that dynamic changes during the initial week in the intensive care unit (ICU) are crucial for evaluating outcomes [22]. These recent data underline the strong prognostic role of dead space and strengthen the rationale for its use in ARDS patients.

#### Corrected minute ventilation

The corrected minute ventilation (*V*_Ecorr_) is a simple and easy-to-calculate surrogate of the dead space fraction that does not require the expired carbon dioxide (CO_2_) measurement. V_Ecorr_ is calculated as the ventilation required to achieve a PaCO_2_ value of 40 mmHg. In mechanically ventilated COVID-19 ARDS patients, Fusina et al*.* found a strong correlation between V_Ecorr_ and the dead space fraction, with a higher *V*_Ecorr_ in nonsurvivors, which was independently associated with mortality [23].

#### Ventilatory ratio

In recent years, in addition to dead space fraction computation, the ventilatory ratio (VR) has been proposed as an additional, easy-to-calculate estimation of ventilation efficiency [24]. VR is computed as the product of minute ventilation and arterial carbon dioxide weighed on the patient’s predicted body weight [24]. It is a unitless ratio, being approximately one in healthy subjects. In ARDS patients, Sinha et al*.* reported a positive relationship between VR and alveolar dead space. Furthermore, VR is more common in nonsurvivors than in survivors [24] and is associated with increased odds of hospital mortality (OR 2.07, confidence interval [CI] 1.53–2.83). As recently shown by Siegel et al*.*, the ventilatory ratio, in association with the APACHE III score at admission, has an area under the curve (AUC) of 0.81 (95% CI 0.68–0.92) in predicting hospital mortality and is significantly better than the APACHE III score alone [25]. Changes in VR within the first 4 h after prone positioning in ARDS patients predict weaning from mechanical ventilation, with an AUC of 0.64 (95% CI 0.53–0.75) [26].

VR reliability can be affected by venous admixture (*Q*_va_/*Q*) and the amount of patient CO_2_ produced (VCO_2_). Indeed, these two factors may increase the difference between alveolar and arterial PCO_2_, with the latter being used for VR calculations. Maj et al*.* showed that the predictive value of the VR decreases in most severe patients, who are affected by greater *Q*_va_/*Q* impairment [27]. To investigate the effect of VCO_2_, Monteiro et al*.* performed a post hoc analysis of the PETAL-ROSE trial [28]. The authors showed that the presence of neuromuscular blockade, a factor influencing skeletal muscle CO_2_ production, did not significantly affect the relationship between VR and mortality [29].

In mechanically ventilated patients, dead space should be continuously assessed as an additional measurement of gas exchange impairment, together with the PaO_2_/FiO_2_ or SpO_2_/FiO_2_ ratio. The use of surrogates, which are easier to calculate, seems to be reliable and should encourage the use of such measures to predict patient outcomes. Caution must be used in moderate and severe ARDS patients with major Q_va_/Q impairment when assessing VR. In these situations, dead space might be overestimated.

#### ETCO_2_ to arterial PCO_2_

A further parameter to estimate gas exchange efficiency is the computation of the end-tidal-to-arterial PCO_2_ ratio (P_ET_CO_2_/PaCO_2_), which measures the influence of venous admixture and alveolar dead space on lung performance. Ideally, this ratio should be equal to one. Bonifazi et al*.* showed that the P_ET_CO_2_/PaCO_2_ ratio significantly decreases from mild to severe ARDS [30]. Additionally, P_ET_CO_2_/PaCO_2_ is strongly correlated with the amount of nonaerated tissue measured via computed tomography (CT) and respiratory compliance [30]. A subsequent study revealed a relationship between the P_ET_CO_2_/PaCO_2_ ratio, alveolar ventilation and hospital mortality [31]. For every 0.01 increase in the P_ET_CO_2_/PaCO_2_ ratio, the risk for mortality decreases by 1%.

Currently, weaning from venous extracorporeal membrane oxygenation (VV-ECMO) lacks well-defined criteria and is often based on acceptable blood gas analysis and the absence of excessive inspiratory effort. In a recent multicenter study, Lazzari et al*.* showed that the P_ET_CO_2_/PaCO_2_ ratio, with a cutoff of 0.83, is able to predict weaning [32].

### Ventilation and patient self-induced lung injury

Mechanical ventilation and spontaneous inspiratory effort may be harmful. The mechanical power, its normalization (i.e., the mechanical power ratio) and the measurement of the esophageal pressure are crucial to minimize these sources of lung injury in patients affected by ARDS. Indices of recruitment are helpful for adequately establishing mechanical ventilation.

### The mechanical power

Mechanical power refers to the energy dissipated in the respiratory system while moving a specific volume at a given PEEP. It is typically expressed in Joules per minute (J/min) [33]. This energy dissipation within the respiratory system plays a crucial role in modulating and potentially promoting ventilator-induced lung injury (VILI). The mechanical power is a unifying indicator computed considering the major ventilatory variables generated from the interaction between the patient and ventilator. It can be assessed under passive conditions and categorized based on ventilation modality (pressure or volume-controlled ventilation) using algebraic equations [34]. The newest intensive care mechanical ventilators now offer the possibility to directly measure mechanical power, with acceptable accuracy compared to traditional algebraic methods [35].

Recent studies have demonstrated that mechanical power at admission is associated with hospital mortality across a heterogeneous range of patients [36–38]. Urner et al. further explored the relationship between the intensity of mechanical power throughout the intensive care stay and mortality, revealing an increased risk of death with each additional day of exposure to mechanical power equal to or greater than 17 J/min [39]. Pozzi et al. analyzed the clinical course of ventilatory variables in ARDS patients during the initial three days of MV and identified the mechanical power ratio at admission as the only variable associated with intensive care mortality [40]. By day 3, the mechanical power ratio, alveolar dead space, and PaO_2_/FiO_2_ were associated with the outcome. Therefore, in ARDS patients, assessing ventilatory variables during the initial days of mechanical ventilation seems to be crucial for predicting outcomes.

Concerning the different components of mechanical power, Costa et al*.* showed a stronger association with mortality for the dynamic component (i.e., the respiratory rate and the driving pressure) than for the total mechanical power [41]. However, the impact of similar values of mechanical power on lung injury can vary significantly based on factors such as ventilated lung size, respiratory system compliance, or the amount of aerated tissue at a given PEEP. Coppola et al*.* demonstrated that normalizing the mechanical power at admission to the compliance of the respiratory system and the amount of ventilated tissue, as computed by lung CT, provides a better predictive measure for outcomes in ARDS patients [36].

### Esophageal pressure and diaphragmatic ultrasound

Preserving spontaneous breathing over invasive ventilation offers advantages [42, 43]. However, elevated inspiratory efforts are associated with high negative esophageal pressure (Pes) swing and positive transpulmonary pressure, which may lead to patient self-inflicted lung injury (PSILI). PSILI is associated with organ dysfunction and increased mortality [44–46]. Additionally, excessive inspiratory effort cannot be detected simply by monitoring airway pressure [47].

Computing the changes in esophageal pressure during inspiration (ΔPes) as the difference between the esophageal pressure at the beginning of inspiration and its lowest value is the easiest way to measure inspiratory effort. In the presence of acute respiratory failure, several noninvasive respiratory support methods, such as HFNC therapy, continuous positive airway pressure (CPAP), and noninvasive ventilation (NIV), should improve gas exchange and decrease inspiratory effort. Menga et al*.*, in a crossover study comparing noninvasive support, showed that only NIV delivered by a helmet is able to reduce delta pes [48]. In a large group of COVID-19 ARDS patients receiving helmet CPAP, total stress, defined as the sum of the transpulmonary pressure generated by the patient and the end expiratory airway pressure, is independently associated with a negative outcome [49].

Transpulmonary pressure measurements allow clinicians to evaluate lung recruitment efficacy. With this aim, it can be useful to evaluate the effects of awake prone positioning, as performed in COVID-19 ARDS patients. Prone positioning leads to a reduction in ventral alveolar hyperinflation and dorsal atelectasis, thus promoting homogenization of transpulmonary pressure and improvement in oxygenation. Additionally, as demonstrated in a cohort of COVID-19 ARDS patients assisted with helmet CPAP, prone positioning significantly reduces the amount of work involved in breathing [50]. The role of esophageal pressure manometry in evaluating inspiratory effort and preventing PSILI is increasingly recognized, and this technique is always recommended for ARDS patients.

Another possible way to evaluate inspiratory effort is the use of ultrasound. However, Steinberg et al*.* show poor correlation between diaphragmatic thickening fraction (DTI), diaphragmatic excursions and esophageal swing in a cohort of 46 mechanically ventilated patients affected by Covid-19 ARDS [51]. Similarly findings are available from Poulard et al*.* [52]. Delta Pes monitoring remains therefore essential to evaluate PSILI in patients undergoing assisted mechanical ventilation.

Nevertheless, diaphragmatic ultrasound remains a valid tool to predict weaning from MV, and recent studies strengthen this evidence. Mawla et al*.* find a possible cutoff of 13.5% for DTI as accurate to predict weaning from MV [53]. Another original investigation shows how the association of different diaphragmatic ultrasound indexes has an area under the curve of 0.77 in predicting extubation success [54].

### Recruitment: the recruitment/inflation ratio and the EIT-based PEEP titration

Chen et al. proposed the recruitment-to-inflation ratio (R/I ratio) as a noninvasive method to compute the potential for lung recruitment at different PEEP levels [55]. Subsequently, the R/I ratio has been clinically validated to be accurate in detecting lung recruitment in ARDS patients in the supine position [56, 57]. In a secondary analysis of a previous study [58], the R/I ratio at two levels of PEEP, both in the supine and prone positions, correlated with lung recruitment computed by CT scan [59]. In addition, the overall data confirm high variability in lung recruitability among ARDS patients, with different effects on gas exchange, respiratory mechanics and hemodynamics. Zerbib et al*.* reported that an R/I ratio > 0.62 predicts lung recruitability with an AUC of 0.80 in COVID-19 ARDS patients [60]. Patients with high recruitability show an improvement in both oxygenation and respiratory system compliance, while in patients with low recruitability, an increase in oxygenation is associated with a decrease in cardiac output. These data confirm that the R/I ratio is a valuable aid for physicians to select an adequate level of PEEP, to improve respiratory mechanics and oxygenation, and to monitor hemodynamics and cardiac output.

New interesting evidences are available about electrical impedance tomography (EIT) as an effective tool to titrate PEEP in patients affected by ARDS. In an original article on 108 Covid-19 ARDS patients, PEEP titration was performed during EIT monitoring, via decrementing PEEP trials [61]. The authors identify the best PEEP as the one corresponding to the crossing point of the collapse–overdistension curves. They also determine the PEEP with the best regional distribution of ventilation. Interestingly, EIT-based PEEP found at the collapse-overdistension crossing point well correlates to the PEEP with the highest compliance, while PEEP with the best EIT ventilation distribution is higher than the previous ones [61]. Jimenez et al*.* show that EIT-based PEEP setting allows to decrease mechanical power in ARDS patients, thus being potentially able to reduce VILI in this population [62]. Robust data on clinical outcomes of PEEP titration techniques are still lacking in the literature. A multicenter randomized controlled trial is actually going on to find out differences on clinical outcomes in ARDS patients whose PEEP is titrated using either EIT-based techniques or PEEP/FiO_2_ tables [63].

### Cardiac monitoring

In ARDS patients, hemodynamic instability and low cardiac output may further decrease oxygen delivery and promote tissue hypoxia [64]. Strategies aimed at increasing cardiac output often involve fluid administration and vasoactive agents [65]. Therefore, hemodynamic monitoring is crucial in ARDS patients to optimize fluid administration and cardiac output [66].

#### Dynamic indexes of fluid responsiveness

As also recently highlighted by the Surviving Sepsis Campaign, the intravenous fluids of choice for critically ill patients are balanced crystalloids [67]. The risks of net fluid accumulation in critically ill patients have also recently been advocated [68]. The deliberate choice of a liberal versus a restrictive fluid strategy fails to show benefits in terms of reducing mortality [69]. A possible decrease in terms of length of ICU stay and mechanical ventilation is demonstrated in patients receiving lower amounts of intravenous fluids [70]. These considerations underline the importance of fluid administration optimization in mechanically ventilated patients.

Pulse pressure variation and stroke volume variation (PPV, SVV) are established predictors of fluid responsiveness and are typically validated in patients ventilated with a tidal volume greater than 8 mL/kg. Indeed, as also recognized by the acronym LIMITS (low heart/respiratory rate ratio, irregular beats, mechanical ventilation at low tidal volume, increased abdominal pressure, thorax opening, spontaneous breathing), mechanical ventilation at low tidal volume may reduce the sensitivity of these tests [71]. This is a possible limitation for their application in patients ventilated with protective strategies (i.e., ARDS) [72]. However, Wang et al*.* recently demonstrated good PPV performance in patients ventilated with less than 8 mL/kg TVc [73]. Similarly, as highlighted in Table 1, Taccheri et al*.* [74] showed that, in patients ventilated with low tidal volume (6 mL/kg), a PPV or SVV increase of 20% or 1 point after the application of a TVc compared to the baseline is a good predictor of fluid responsiveness in patients ventilated with a low tidal volume.

**Table 1:** Recent evidences on predictors of fluid responsiveness suitable for mechanically ventilated patients

Predictor	Mechanical ventilation settings	Diagnostic cutoff	Recent evidences
PPV	Tidal volume > 8 mL/kg of IBW	13% (*gray zone* between 9 and 13%)	In patients ventilated at low tidal volume (< 8 mL/kg), a TVc increases PPV performance [73]
ΔSVV	Patients ventilated with low tidal volume and application of a TVc (from 6 to 8 mL/kg of IBW)	Increase of 20%^a^ or + 1 point^b^ compared to the values before TVc	AUC of respectively 0.94 and 0.98 in predicting fluid responsivity from Taccheri et al*.* [74]
ΔPPV	Patients ventilated with low tidal volume and application of a TVc (from 6 to 8 mL/kg of IBW)	Increase of 20%^c^ or + 1 point^d^ compared to the values before TVc	AUC of respectively 0.82 and 0.94 in predicting fluid responsivity from Taccheri et al*.* [74]
PLR	Independently from MV parameters	CO increase of 5% or EtCO_2_ increase of 5%/2 mmHg or PPI increase of 9%	+ 4% of ΔScvO_2_ after a PLR validated by Giraud et al. with an AUC of 0.92 [78]
ΔIVC	Patients ventilated with low tidal volume (6 mL/kg)	+ 4% of ΔIVC after a TVc^e^ or − 24% after a PLR^f^	AUC of respectively 0.76 and 0.88 in predicting fluid responsiveness from Taccheri et al. [74]
PEEP test	PEEP decrease from 10 to 5 cmH_2_O	Increase of CI > 8.6% when compared to the baseline	AUC of 0.94 in predicting fluid responsiveness [75]

Recent evidences about predictors of fluid responsiveness in mechanically ventilated patients

*PPV* pulse pressure variation, *IBW* ideal body weight, *TVc* tidal volume challenge, *mL* milliliters, *kg* kilograms, *ΔSVV* relative changes of stroke volume variation, *ΔPPV* relative changes of pulse pressure variation, *AUC* area under the receiver operating characteristic curve, *PLR* passive leg raising, *MV* mechanical ventilation, *CO* cardiac output, *EtCO*_*2*_ end-tidal carbon dioxide, *PPI* plethysmographic peripheral perfusion index, *ΔScvO*_*2*_ changes in central venous oxygen saturation, *ΔIVC* changes in inferior vena cava diameter, *PEEP* positive end expiratory pressure

As recently reported by Lai et al*.*, in patients mechanically ventilated with high positive end expiratory pressure (> 10 cmH_2_O), a decrease in PEEP (the so-called PEEP test) is a possible alternative to passive leg raising (PLR) to demonstrate fluid responsiveness. The authors have shown that an increase in the cardiac index is evident after both a PLR or with a decrease in PEEP from 10 to 5 cmH_2_O, with high sensitivity and specificity [75] (Table 1). According to Perez et al*.*, the validity of these results has to be further proven, as indicated by the low respiratory compliance of the study population in the original paper [76].

#### Central venous oxygen saturation

Central venous oxygen saturation (ScvO_2_) is a valuable indicator of oxygen delivery adequacy for patients who are not equipped with a pulmonary artery catheter and for whom mixed venous oxygen saturation (SvO_2_) is not available. Concerns have been recently raised about its reliability during mechanical ventilation: it has been shown that the difference between ScvO_2_ and SvO_2_ may increase when intrathoracic pressures grow, especially when high PEEPs are employed [77]. Nevertheless, beyond the ScvO_2_ absolute values, its variation responding to diagnostic maneuvers may provide useful hemodynamic insights. A recent prospective study showed that the increase in ScvO_2_ (ΔScvO_2_) after PLR is a good predictor of fluid responsiveness and is associated with an increase in cardiac index [78]. These findings and the cutoff found by the authors (+ 4%) are comparable to the results of a meta-analysis from Pan et al*.*, in which the authors showed that ΔScvO_2_ after a fluid challenge (500 mL) may adequately predict fluid responsiveness [79]. Another possible indicator of fluid responsiveness and cardiac output adequacy is the veno-arterial carbon dioxide difference (Pv-aCO_2_), together with the arterial to venous oxygen content difference (Ca-vO_2_) [80]. In COVID-19 patients with ARDS, the ratio of Pv-aCO_2_ to Ca-vO_2_ was significantly associated with mortality, with an AUC of 0.89 (95% CI 0.598–0.774, P = 0.001). The best cutoff found by the authors was 2.1 mmHg/mL [81].

#### Ultrasound and fluid responsiveness

The use of the Velocity Time Integral (VTI) of the Left Ventricular Outflow Tract (LVOT) allows a semi-continuous measure of the stroke volume (SV) and its variation (ΔSV) after a fluid challenge or a passive leg raising. It is therefore a valuable and well-validated measure for evaluation of fluid responsiveness [82]. Recently, it has been demonstrated how LVOT-VTI time variation well correlates with other parameters of fluid responsiveness, such as PPV, in a cohort of surgical patients [83]. However, LVOT-VTI measure is not always easy to assess and might be influenced by inter-operator variability [84]. The measure of the carotid systodiastolic (CSD) flow has been recently proved to be a valid surrogate of the LVOT-VTI, easier to measure [85]. Further researches may be useful to validate this parameter and allow its use to evaluate fluid responsiveness.

Respiratory change in inferior vena cava diameter (ΔIVC) is easy to measure with little expertise on thoracic ultrasound. It may provide important information on cardiac preload [66]. However, its sensibility is lower in patients ventilated at low tidal volume and in spontaneously breathing patients [74]. In their report, Taccheri et al*.* show that, similarly to PPV and SVV, ΔIVC is potentially applicable to patients ventilated at low tidal volume, thus enhancing their applicability on ARDS patients. The authors find an AUC of 0.76 and 0.86 of ΔIVC in predicting fluid responsiveness, respectively, after a TVc or a PLR when the applied tidal volume is less than 6 mL/kg (Table 1) [74].

#### Capillary refill time

The capillary refill time (CRT) is a simple-to-evaluate index of peripheral tissue perfusion and microcirculation. Monitoring CRT in critically ill patients reduces organ dysfunction and mortality compared to monitoring lactate levels alone [86]. In a prospective observational study, Raia et al*.* showed a direct correlation between a decrease in CRT after a 500-mL fluid challenge and fluid responsiveness. A low percentage (0.5%) of the total variance of the measurements is due to operator dependence (intrareader variability), thus stressing the reliability of this noninvasive parameter for assessing fluid responsiveness [87]. Caution must be taken in the use of CRT in patients with vasodilatory shock. Fage et al*.* showed that in septic patients, the correlation between a decrease in CRT and fluid or vasoactive administration is consistent only when substantial increases in the mean arterial pressure (MAP) or cardiac index (CI) are recorded. In contrast, in patients with an increase in MAP and CI less than 15% compared to the baseline, CRT is poorly correlated with fluid and vasoactive responsiveness [88]. CRT is an indirect index of microcirculatory dysfunction. In 282 critically ill patients, a correlation between CRT and microcirculatory impairments has been highlighted using the *sidestream dark field imaging* technique. CRT is independently correlated with the microvascular flow index (MFI). Patients admitted to the ICU with a higher CRT have a higher mortality [89]. Similar findings have been described in patients with COVID-19-related ARDS, in which, despite hemodynamic stability and normal lactate levels, CRT and microcirculatory indices (such as the MFI) are impaired, resulting in altered tissue perfusion [90].

CRT ranks therefore among the useful and easily measurable parameters to assess fluid responsiveness, and its use must be encouraged, with a word of caution in patients with vasodilatory shock. In the latter, monitoring CRT to test fluid responsiveness may result in false negatives due to decreased test sensitivity.

## Conclusions

In patients affected by ARDS, harmful mechanical ventilation associated with a positive fluid balance may worsen lung injury. Careful respiratory and hemodynamic monitoring is therefore crucial in these patients.

The SpO_2_/FiO_2_ ratio is a valid alternative to the PaO_2_/FiO_2_ ratio for evaluating gas exchange. Corrected minute ventilation and the ventilatory ratio are two valuable surrogates for estimating the dead space fraction, and their prognostic value is well recognized in ARDS patients. Mechanical power directly measures the energy delivered to the lungs by mechanical ventilation and is thus able to predict VILI in invasively ventilated patients. It is easy to calculate because algorithms can be directly implemented in mechanical ventilators. In patients undergoing noninvasive mechanical ventilation, the role of esophageal pressure is crucial for estimating the P-SILI.

In addition to respiratory variable monitoring, fluid stewardship is also important for detecting VILI. Dynamic indices of fluid responsiveness are also suitable for patients undergoing protective mechanical ventilation at low tidal volumes. There is increasing evidence about the validity of the ΔscvO_2_ and CRT after a PLR or a fluid challenge. The role of these easy-to-evaluate parameters may be increasingly important in patients affected by respiratory failure, especially in disadvantaged contexts in which enhanced monitoring is not available.

## Data Availability

Data sharing is not applicable to this article, as no datasets were generated or analyzed during the current study.

## Abbreviations

ARDS: Acute respiratory distress syndrome
PEEP: Positive end expiratory pressure
MV: Mechanical ventilation
SaO_2_: Arterial oxygen saturation
SpO_2_: Oxygen saturation measured via pulse-oximetry
SpO_2_/FiO_2_: Pulse-oximetric oxygen saturation to the fraction of inspired oxygen
PaO_2_/FiO_2_: Partial pressure of arterial oxygen to FiO2
COVID-19: Coronavirus disease
HFNC: High-flow nasal cannula
RCT: Randomized controlled trial
*V*/*Q*: Ventilation/perfusion
ICU: Intensive care unit
*V*_Ecorr_: Corrected minute ventilation
CO_2_: Carbon dioxide
VR: Ventilatory ratio
AUC: Area under the curve
*Q*_va_/*Q*: Venous admixture
*V*CO_2_: Amount of CO_2_ produced
PETCO_2_/PaCO_2_: Gas exchange efficiency is the computation of the end-tidal-to-arterial PCO2 ratio
CT: Computed tomography
VV-ECMO: Venous extracorporeal membrane oxygenation
J/min: Joules per minute
VILI: Ventilator-induced lung injury
Pes: Esophageal pressure
PSILI: Patient self-inflicted lung injury
Δpes: Changes in esophageal pressure during inspiration
CPAP: Continuous positive airway pressure
NIV: Noninvasive ventilation
DTI: Diaphragmatic thickening fraction
*R*/*I* ratio: Recruitment-to-inflation ratio
EIT: Electrical impedance tomography
PPV: Pulse pressure variation
SVV: Stroke volume variation
LIMITS: Low heart/respiratory rate ratio, irregular beats, mechanical ventilation at low tidal volume, increased abdominal pressure, thorax opening, spontaneous breathing
TVc: Tidal volume challenge
PLR: Passive leg raising
ScvO_2_: Central venous oxygen saturation
SvO_2_: Venous oxygen saturation
ΔscvO_2_: Central venous oxygen saturation increase
Pv-aCO_2_: Veno-arterial carbon dioxide difference
Ca-vO_2_: Venous oxygen content difference
VTI: Velocity time integral
LVOT: Left ventricular outflow tract
SV: Stroke volume
ΔSV: Variation in stroke volume
CSD: Carotid systodiastolic
ΔIVC: Change in inferior vena cava diameter
CRT: Capillary refill time
MAP: Mean arterial pressure
